# A systematic review regarding women’s emotional and psychological experiences of high-risk pregnancies

**DOI:** 10.1186/s40359-020-00410-8

**Published:** 2020-05-03

**Authors:** Nazeema Zainura Isaacs, Michelle Glenda Andipatin

**Affiliations:** Department of Psychology, University of the Western Cape, Cape Town, South Africa

**Keywords:** Systematic review, Qualitative methods, High-risk pregnancy, Maternal health, Emotional/psychological experience, Severe morbidity, Medical conditions and complications

## Abstract

**Background:**

High-risk pregnancy refers to a pregnancy that negatively affects the health of the mother, the baby, or both. High-risk pregnancy evokes a range of emotional and psychological experiences for the expectant mother, and can adversely affect both the mother and the baby’s health. Medical research on high-risk pregnancy abounds, while women’s emotional/psychological experiences are not sufficiently documented, and hence much less attention and/or programming is directed to support women with high risk pregnancies.

**Methods:**

The aim of this review is to present published evidence of how studies reported on the emotional and psychological experiences of a woman’s high-risk pregnancy journey. The systematic review examined qualitative studies over a 10 year period that were published between January 2006 and June 2017. These studies were identified on 10 databases. The study utilised three stages of review (i.e. abstract reading, title reading, and full-text reading) and for a successful conduction of the meta-synthesis, this study applied one of the phases provided by Noblit and Hare.

**Results:**

The findings provide empirical evidence that women’s emotional and psychological experiences (i.e. shock, fear, frustration, grief, isolation and loneliness, anger, sadness, guilt, and mental health disorder) are evident throughout their high-risk pregnancies experience.

## Background

After decades of improvement, there are still maternal and infant deaths [1]. Koblinsky, Chowdhury, Moran, and Ronsmans [2] argue that 15% of all pregnancies will develop into life-threatening complications. During 2013, there were 289,000 maternal deaths, of which sub-Saharan Africa accounted for 60% and South Asia 24% [3]. However, between 1990 and 2015, global maternal deaths per 100,000 live births decreased by 2.3% per year, specifically in regions such as Asia and North Africa [3]. According to the national department of health (NDoH) in the annual performance plan of 2013/2014 report, the maternal mortality ratio is 310 per 100,000 live births which is double than the previous year in South Africa [4]. A large percentage of these maternal deaths can be attributed to high-risk pregnancy complications.

A high-risk pregnancy is defined as “any pregnancy in which there is a medical factor, maternal or foetal that potentially acts adversely to affect the outcome of pregnancy” [5]. Platt et al. [6] argues that a pregnancy could be complicated in three ways. Firstly, complications can be a result of abnormal responses in the mother’s body to the pregnancy-induced changes that greatly affect the health of the baby. Secondly, complications may stem from atypical development such as serious abnormalities and genetic or congenital disorders that occur in the baby. Lastly, medical conditions associated with labour and delivery, including preterm labour, gestational diabetes, preeclampsia, and placental previa complicate a pregnancy.

Despite the fact that the emergence/y of modern obstetrics can be linked to the 1950s, the term ‘high-risk pregnancy’ was not employed [7]. During the 1960s, literature started referring to the concept of high-risk pregnancy [8]. Roderigues et al. [9] reveal that healthcare providers consider the label of high-risk pregnancy to be an indication that a woman and/or her unborn infant are at risk of both physical and psychological harm.

When expectant women are diagnosed with a high-risk pregnancy, they may have difficulty confronting and dealing with this new reality which results in psychological and emotional consequences [10]. The literature reveal that women who are experiencing and have experienced high-risk pregnancies have a host of emotional issues including fear, guilt, shock, grief, frustration, worry, loneliness and isolation [11, 12]. Currie and Barber [13] contend that when there is a health threat during the pregnancy, women are more likely to experience psychological distress. Similarly, Simmons and Goldberg [14] report that the label ‘high-risk’ pregnancy is associated with higher psychological distress. Some women may experience either the onset or relapse of some serious psychological disorders [15]. Psychological disorders during pregnancy may result in low birth weight and preterm delivery, and women with bipolar disorder experience onset of mood instability [16, 17]. When women are diagnosed with postpartum onset of major depression disorder they may have obsessions of child harm, and suicidal thought [13]. Therefore, not all pregnancies are simple, straightforward events.

### Rationale

Previous research on high-risk pregnancies primarily focused on the medical aspects and the emotional and psychological experiences are not acknowledged sufficiently. Recently, various worthwhile studies found in Europe, Africa, and North America have reported on the medical aspects and emotional and psychological experiences of women who experienced high-risk pregnancies [14, 15, 18, 19]. These studies have revealed that there exist a clinical relationship between the medical aspects as well as emotional and psychological challenges during a women’s high-risk pregnancy journey and more studies should explore this simultaneously. The objective of the study is to examine the emotional and psychological experiences of women throughout their high-risk pregnancies. Additionally, to present evidence that medical issues, emotional and psychological experience of a pregnant woman must be studied together to obtain a holistic understanding of a women’s high-risk pregnancy journey.

## Method

This study utilised a systematic review defined as a review that uses systematic and explicit methods to identify, select, and critically appraise relevant research; to collect and synthesise data from the studies included in the review; and to interpret the findings [20, 21]. Additionally, it intends to diminish bias in selection. This methodology was considered the most suitable method of rigorous review because it evaluates and summarises studies across the world on women’s emotional and psychological experiences of high-risk pregnancies. This method will allow the researcher to critically appraise and scrutinise the findings of the studies that explored this topic. The format of the Method section in this paper was guided by a colleague who previously conducted a systematic review.

### Inclusion criteria

The period of January 2006 to June 2017 was selected to reflect on the more recent literature within this specific field and given that it was information based on a Master’s thesis. The review included studies where women spoke about their emotional and psychological experiences throughout their high-risk pregnancies in their lifetime. The review considered studies that used qualitative data collection methods and analysis, and included the qualitative component of mixed method studies. Studies were also limited to the English language. Lastly, studies that required payment were included as the University of the Western Cape (UWC) has a subscription with several databases that made them available to registered students.

### Exclusion criteria

Studies were excluded based on the following criteria: articles that focus on childbearing women in general, the full report/text is not available, the manuscript is not published in English, it does not target the desired population, it reports on a quantitative study, or it was written and published before January 2006 and after June 2017. There was no grey literature added in the study because of the methodological challenges when it came to critically appraising it and not having the same format as an academic article.

### Review process

The review process for the present study involved four steps. The first step (i.e. identification) consisted of identifying and retaining potential studies that could be included in the review. This involved three actions; first, keywords had to be identified. Secondly, the researcher did a thorough search of the databases at UWC by using the keywords and index terms identified. A selection of 10 databases included EbscoHost, JSTOR, Sage Journals Online, ScienceDirect, SpringerLink, Sabinet, Scopus, Emerald eJournals Premier, PubMed as well as Taylor and Francis Open Access eJournals. Lastly, the researcher consulted sources such as cross-referencing and made use 16 other sources. However, five of these studies that were cross-referenced by the researcher and speak to the research questions of this study were included. The second step (i.e. screening) was the abstract level assessment. The abstracts of the articles that were included after the title search step were assessed and screened based on the inclusion criteria.

The third step (i.e. eligibility) is implemented whereby the articles that had been included based on their abstracts and the fact that they attempt to answer the research questions of this review, were screened for methodological rigour utilising a critical appraisal tool. The critical appraisal tool utilised in this study was developed by Smith, Franciscus, Jacobs, Munnik, and Swartbooi (under review) [22]. The critical appraisal tool allowed for each article to be scored. In this review, the threshold score for inclusion was set at strong (61–80%). Articles that attained a score below 61% was deemed weak and excluded from the review. Setting a high threshold score was appropriate, as it would enhance the study without affecting its comprehensiveness. For this study, 17 articles were included in the review of which, seven scored between 61 and 79%, and ten articles scored in the excellent range (> 80%), of which the highest rated article scored 97% indicated in Table 1 below.

**Table 1:** Ranking according to the critical appraisal tool

1	Saukko (2009) [23]	97%	> 80% (excellent)
2	Roomaney, author, & Naidoo (2014) [15]	96%	
3	Ncube, Barlow, & Mayers (2016) [24]	93%	
	Norhayati, Asrenee, Hazlina, & Sulaima (2017) [25]	93%	
4	Tinoco-Ojanguren, Glantz, Martinez-Hernandez, & Ovando-Meza (2008) [26]	91%	
5	Greenhalgh et al. (2015) [27]	84%	
	Kaye et al. (2014) [28]	84%	
6	Lalor, Devane, & Begley (2007) [29]	82%	
	Price et al. (2007) [30]	82%	
7	Lalor & Begley (2006) [31]	80%	
8	Lalor, Begley, & Galavan (2009) [32]	76%	61–79 (strong)
9	Khan, Bilkis, Blum, Koblinsky, & Sultana (2012) [33]	73%	
10	Souza, Cecatti, Krupa, Osis, & Parpinelli (2009) [34]	71%	
11	Alex & Whitty-Rogers (2017) [35]	69%	
12	Curran, McCoyd, Munch, & Wilkenfeld (2017) [36]	64%	
13	Malouf & Redshaw (2017) [37]	62%	
	Yeakey, Chipeta, Taulo, & Tsui (2009) [38]	62%	

Articles appraised and selected for retrieval were assessed by two independent reviewers for methodological rigour. The two reviewers were included to avoid bias. It is considered a convention in systematic reviews. The second reviewer is a PhD intern at UWC with experience in conducting systematic reviews. He was briefed on the methodology and all the steps involved in the process. Both reviewers consulted the necessary literature to familiarise themselves with the process. Working together in pairs enabled verification and allowed the supervisor to act as a control with the final say on all the decisions made at the various phases in the review process. The benefit of working in pairs was that it allowed the verification to contribute greatly to the highest possible level of methodological rigour for this research study. Any disagreements between the two reviewers were resolved during a discussion and the supervisor acted as a third party control to help the reviewers reach consensus. However, a disagreement emerged during the critically appraisal stage as different scores were given on two articles and this was resolved as both reviewers came to an agreement. There were not many disagreements during the critical appraisal phase. All disagreements can be considered as minor and were resolved through a discussion.

The last step of the review process is summation whereby data was extracted from the 17 included studies and this was done through a process called meta-synthesis. For a successful conduction of the meta-synthesis, this study utilised one of the phases provided by Noblit and Hare [39]. The phase that was utilised was the reciprocal stage and it refers to how each of the articles selected relate to each other [39]. Additionally, it consists of identifying frequent themes and interests found in the selected articles that relate to answering the research questions. The main activity in this phase is highlighting themes that are prevalent in more than one of the included studies. Part of this phase consisted of the researcher constantly reading all the articles selected for inclusion in the current study. As the researcher read these studies, highlighted and documented all the emotional and psychological experiences discussed in the articles. In addition, the researcher made a list of the articles that mentioned the same emotional and psychological experiences. Once this process was completed for each article, the researcher then compared and placed the list of emotional and psychological experiences, alongside the existing literature. Afterwards, the researcher consulted the findings of the included articles and the findings of the existing body of literature to identify similarities. These findings is thoroughly explored in the Results section.

**Fig. 1:**
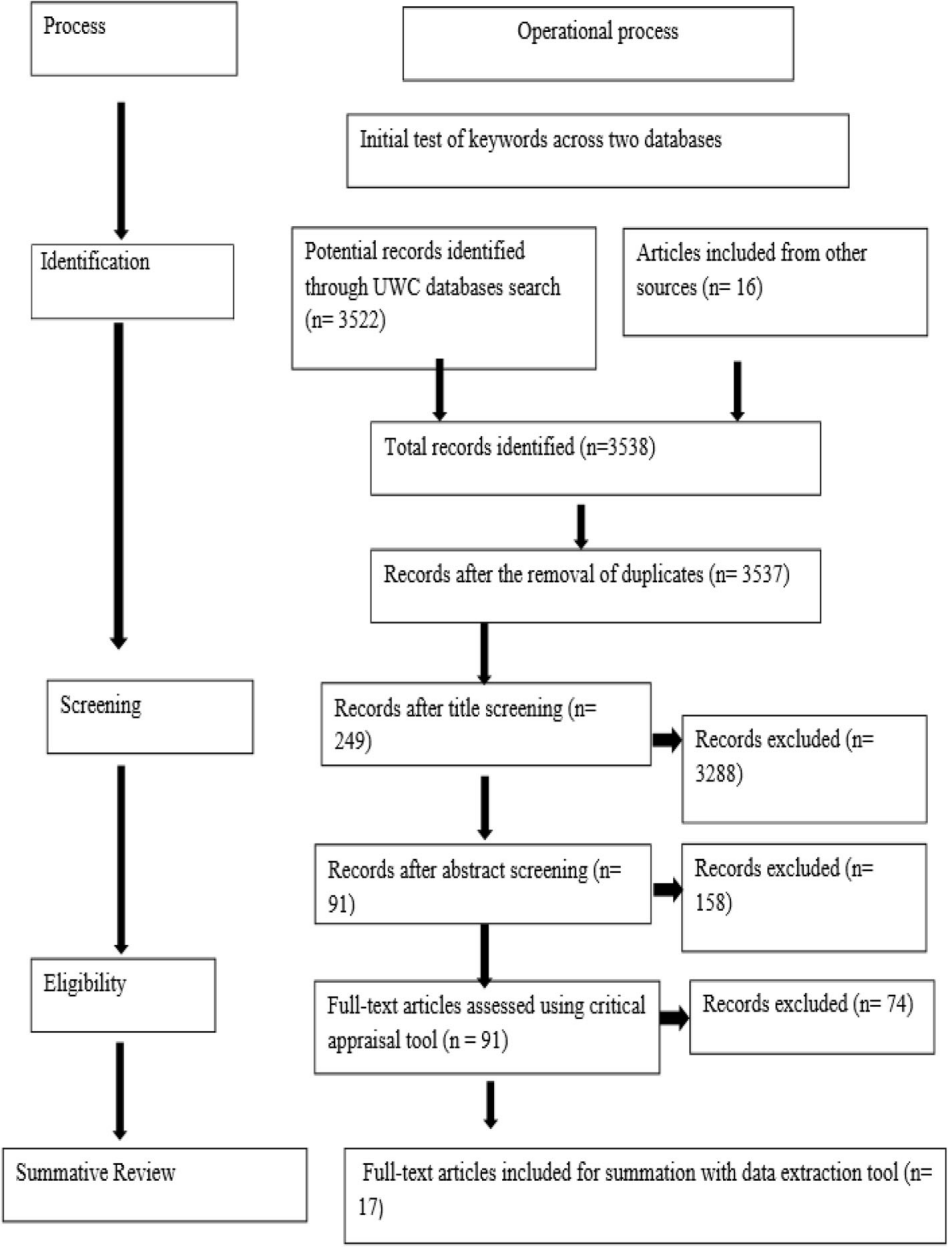
Review Process Results

The results of the review process are represented graphically in Fig. 1 below. The researcher has adapted the recommended PRISMA flow chart in Moher et al. [40]. The numbers in brackets refer to the quantity of articles identified by electronic database searching.

## Results

### General description of the studies characteristics

The table below provides the general description of the studies included in this review. All 17 articles discussed at least one or more emotional/psychological experiences of women throughout their medically complicated pregnancies (Table 2).

**Table 2:** General description of the studies characteristics

Geographical locations	Theoretical orientation	Design	Data Collection	Data Analysis
Ireland, United States, England, South Asia, Bangladesh, Brazil, Canada, Haiti, Uganda, Botswana, Malaysia, Mexico, South Africa, Malawi.	grounded theory, phenomenology, symbolic interactionism, and social network theory	qualitative methodology	focus groups and individual interviews	constant comparative method, thematic analysis, and phenomenological analysis, content analysis, grounded theory analysis,

### Emotional and psychological experiences

The included articles seem to suggest that there are core themes that underpin the high-risk pregnancy experience for women. It often start at a diagnosis of pregnancy-related complications during an ultrasound examination which is traumatic for an expectant mother, as most pregnant women anticipate that their foetus would be healthy and they do not perceive themselves to be at risk [32]. Women diagnosed with a high-risk pregnancy are hospitalised for a prolonged period of time to ensure effective management of their condition and to monitor the growth and well-being of the foetus. Hospitalisation can be stressful for pregnant women because their environment is disrupted and this create a range of emotional responses expressed by pregnant women at risk. The painful aftermath of a traumatic experience is further complicated by the insufficient support that these pregnant women often receive [33]. Consequently, the traumatic experience creates a range of emotions within the expectant women and the emotions aligned with this experience are discussed below.

#### Shock

Shock is defined as having a physical reaction to a recognised wound that does not resolve [41]. Participants in the included studies experienced the emotion of shock at the diagnosis and after giving birth. The experience of shock at the diagnosis was reported in four of the included studies. These studies were conducted in United States, Ireland (*n* = 2), and Canada which indicate that expectant mothers were shocked after being informed by medical staff of their high-risk pregnancies [30, 31, 42, 43]. The participants in the reviewed articles had difficulty internalising the idea that they are suffering from a high risk pregnancy because of the trauma they experienced. The findings of these studies coincided with the report of Lawson and Rajaram [42], who report that their participants’ experienced emotional shock after the diagnosis of a high-risk pregnancy. For this reason, the suddenness of being placed into medical care resulted in the expectant women to experience dread and astonishment.

Further to this, participants’ were unprepared for the outcome of the high-risk pregnancy. In two of the included articles that were conducted in Botswana and Malaysia, participants who suffered from maternal near-miss gave birth to preterm infants and stated that they were shocked [29, 44]. These findings were consistent with the literature as Storeng and colleagues indicated that after the delivery of the infant from a high-risk pregnancy, the mothers still experienced the emotion of shock [45] and had difficulty caring for the infant. This is understandable because mothers faced challenges in pregnancy that resulted in them reliving the shock.

#### Fear

Fear is defined as a mood state with strong negative affect that encourages one to avoid danger [46]. Participants in the included studies experienced fear throughout the whole pregnancy and it is discussed below.

Several facets of pregnancy are unknown and there is an aura of uncertainty that may accompany the pregnancy [38]. The findings of three of the included articles carried out in Botswana, Malawi, and Ireland assert that fear of the unknown dominated the expectant women after being diagnosed with high-risk pregnancy conditions (i.e. foetal abnormality and preterm birth) [29, 47, 48]. The uncertainty of the actual health and development of the foetus created the vagueness for the expectant mother and this culminated in the fear for the unknown. In Souza’s et al. [34] study in Brazil, participants feared that their infants’ health would deteriorate after experiencing maternal near-miss. In addition, Ncube et al. [24] acknowledge that participants in a study conducted in Botswana feared for the survival of their new-born infant and expressed how they dreaded handling the tiny infant because they were afraid of ‘damaging’ the new-born infant. These fears were so intense and appeared to be associated with potentially harming their infants. The trepidation expressed by participants from South Africa and Ireland in the respective studies demonstrate the fear associated with attaching it to the baby and how this would escalate the emotional pain should the infant pass away [15, 31]. Thus, fear of foetal death was a common thought expressed by the participants who experienced traumatic births within their hospital stay. This is because the infant was physically at risk.

For participants who suffered from maternal near-miss during their pregnancies, fear was associated with the complications of life-saving procedures. In the study conducted by Norhayati et al. [25] in Malaysia, some of the participants feared that they would receive blood contaminated with the human immunodeficiency virus (HIV) while others feared the possibility of not being able to conceive in the future after undergoing salpingectomy procedure for their ruptured tubal pregnancy. Salpingectomy is a surgical procedure that includes partial or complete removal of the fallopian tube to treat ectopic pregnancies [49]. The fear of being hospitalised and being operated on was so severe for some participants in studies conducted in Ireland and Brazil that they declined critical procedures required to manage their high-risk pregnancies [37, 48]. Those participants living in Brazil and Malaysia who agreed to medical interventions reported that they had to subject themselves to life-changing procedures that related to a sense of death [37, 44]. High-risk pregnancy affects the women’s physical body. Participants experienced adverse bodily changes that were feared and dreaded. For example, Khan et al. [33] reported that participants in their study that took place in Bangladesh feared that they would experience pain during urination and defaecations and they reported that their bodies changed colour because of the obstetric complications. In addition, participants in Souza’s et al. [34] study in Brazil who experienced maternal near-miss revealed that their physical experience changed such as their belly swelled up a lot and it did not look the same as before. Participants in Norhayati’s et al. [25] study in Malaysia expressed fear of going to the bathroom unattended because they had just experienced a traumatic childbirth and experiencing a weak body was unfamiliar to them. For this reason, participants expressed fear towards the changes of their physical bodies.

#### Frustration

Frustration is defined as an irritable distress in relation to failure [50]. During this review, participants expressed frustration when diagnosed with a medically-complicated pregnancy as well as during hospitalisation and this is described below:

During the ultrasound examination, expectant women felt frustrated when the diagnosis of an abnormality was raised. Three of the included studies conducted in Ireland, Uganda, and Brazil indicate that participants who were diagnosed with a pregnancy complication (i.e. uterine rupture-life threatening obstetric complication, maternal near-miss or foetal abnormality) expressed feelings of frustration [37, 48, 51]. These findings regarding participants’ frustration coincided with relevant literature that the diagnosis of a high-risk pregnancy situation often results in frustration for the expectant woman [7].

Expectant women expressed their frustration with inadequate treatment received from medical personnel and with family members for making decisions on their behalf during hospitalisation. For example, participants in the study conducted in Uganda expressed their frustration with their partners and mother’s-in-law who made decisions while they were sedated during a complicated situation or emergency delivery [51]. Similarly, in the studies carried out in Botswana and England during the hospitalisation phase, participants expressed their frustration towards medical professionals who did not provide them with medical support when they were suffering from pregnancy-related complications such as thrombophilia (i.e. a lifetime threat that results in infertility, miscarriages) or preterm birth [28, 29]. Mother’s-in-law and medical professionals are perceived to be protectors to the pregnant women as well as experts of a woman’s pregnancy journey as a result of their experience, knowledge and clinical training [7]. The discrepancies in defining the situation above to the expectant women could have resulted in the frustration they expressed with the actions of the medical personnel and family members. The findings from this review corroborated with the findings of [24] where participants who suffered from a high-risk pregnancy expressed their frustration with the medical practitioners and family members.

#### Grief

Grief is defined as “a natural human response to separation, bereavement or loss, in particular the loss of a loved one” [52]. Participants selected for the respective articles experienced the emotion of grief at the diagnosis of a high risk pregnancy as well as when their infant/(s) has passed away. When the assumption of normality is shattered by an adverse diagnosis, pregnant women in three of the included studies conducted in Canada and Ireland (*n* = 2) describe their initial emotional reaction as one of grief [42, 43, 48]. Participants who had received this diagnosis were distressed due to the possibility that they might lose the baby. These findings were corroborated in existing literature. For instance, expectant women grieved because the diagnosis is conceptualised at the loss of a healthy infant [38]. It is evident that participants expressed grief at the initial point of an adverse diagnosis regarding the health of their developing foetus.

In this category, mothers shared their feelings of loss and grief regarding death of their infants. For instance, Lalor et al. [31] and Souza et al. [34] indicated that participants in studies conducted separately in Ireland and Brazil expressed grief at the loss of their new-born baby. These findings were similar to those of Krueger [53] who reported that grieving for the loss of an infant is part of the human experience. Therefore, the reminiscence of the loss may eternally rest within the women when they see other expectant women or live babies.

#### Isolation and loneliness

Isolation refers to the absence of close companionship and loneliness refers to the disconnection from others [54, 55]. Isolation and loneliness was experienced when the expectant mother was hospitalised and after the women have given birth. For example, participants in two of the included articles conducted in South Africa and Uganda felt isolated after being hospitalised for uterine rupture or HELLP syndrome [15, 44]. The findings concurred within existing literature presented [5, 25]. These authors report that expectant women who are hospitalised during their high-risk pregnancy felt isolated from their companions. However, constant communication with family members was found to relieve the feeling of isolation and loneliness in the reviewed studies carried out in Botswana and England [28, 29]. Social support is important for any medical event such as a high-risk pregnancy as it might offer expectant women a sense of comfort and belonging [56].

The emotion of isolation and loneliness was mainly a result of being separated from partners and babies after childbirth. For instance, Roomaney et al. [15] and Kaye et al. [28] reveal that participants who experienced uterine rupture or HELLP syndrome in studies that were carried out in South Africa and Uganda felt isolated and alone after the delivery of the infant. As most complicated pregnancies result in the infant being placed in the Intensive Care Unit (ICU) or death of the baby, this could account for the isolation and loneliness that the mothers experienced. Similarly, caregivers or partners of the participants would have to resume their responsibilities after the expiration of paternity leave, which might have compounded the women’s experience of isolation and loneliness. Research has shown that both isolation and loneliness are associated with illness and mortality [57]. For this reason, a medically complicated pregnancy that places the infant at risk of mortality would cause the mother to feel isolated and alone.

#### Anger

Anger is defined as a negative feeling associated with particular cognitive and perceptual distortions and deficiencies [58]. The emotion of anger was expressed by expectant women in two of the included studies during hospitalisation. Participants who were hospitalised for HELLP syndrome or preterm birth in studies that occurred in South Africa and Haiti respectively expressed anger and resentment at the unjust treatment they received from medical professionals during hospitalisation [15, 59]. This emotion of anger emerged from the participants whose condition deteriorated as a result of the negligence of the medical staff as opposed to being mediated by caring responses. One example is the lack of information that participants received from medical personnel. This is consistent with the findings of [45], who reported that their study participants’ expressed anger towards medical practitioners for receiving ill treatment that could have further complicated their pregnancies. The reminiscence of the emotion of anger experienced by the participants was supported by the literature.

#### Sadness

Sadness is an emotion that is triggered by loss [60]. This theme describes the emotion of sadness for mothers during the aftermath experience of pregnancy. Participants’ in studies conducted in United States and Malawi revealed that they had an inability to interact with their counterparts and it resulted in them sobbing which was an expression of their deep sadness [30, 47]. Specifically in Norhayati’s et al. [25] study in Malaysia, when participants who experienced maternal near-miss were informed of their inability to bear more children, they were extremely sad. After a traumatic childbirth, participants in studies carried out in several countries (i.e. United States, Ireland, Uganda, Brazil, and Malaysia) who lost their babies indicated that they were not provided with sufficient reasons for the cause of death, which upset them [30, 37, 43, 44, 51]. Furthermore, Yeakey et al. [38] reveal that participants in their study that were carried out in Malawi who suffered from obstetric fistula felt sad after being informed of their limited ability to fulfil marital roles. These findings were corroborated by a report of Andipatin [7] who reported that after the high-risk pregnancy mothers’ experienced intense sadness.

#### Guilt

Guilt is defined as a sense of responsibility for the harmful actions of another or feeling of having done wrong [61]. This category describes the guilt experienced by mothers after the delivery of the child. The findings from this review showed that the guilt is closely associated with self-blame. Roomaney et al. [15], Souza et al. [34], Malouf and Redshaw [37], as well as Khan et al. [33] indicate that participants who had HELLP syndrome, maternal near-miss or experienced preterm birth in studies conducted in several countries (i.e. South Africa, Brazil, United States, and Bangladesh) felt guilty for a number of reasons, which include but not limited to ignoring important signs during their pregnancy, not strong enough to carry a baby to full term, as well as for falling pregnant and starting families late. These findings coincide with the reports of Amorim et al. [51], as well as Lawson and Rajaram [42] that the guilt experienced by mothers’ increases after a high-risk pregnancy experience. As the identity of a mother includes accepting responsibility for the well-being of the foetus and outcome of their pregnancy, the mothers in a study that took place in United States therefore internalised the guilt they felt which resulted in self-blame [30, 53]. However, some studies in this review reveal that the blame was transferred onto someone else. According to Curran et al. [36], Kaye et al. [28], and Norhayati et al. [25], participants who formed part of three studies carried out in United States, Uganda and Malaysia placed the blame onto private health professionals for their high-risk pregnancy condition (i.e. maternal near-miss or uterine rupture). These findings were similar to that of Kidner and Flanders-Stephans [49], who report that women felt betrayed by healthcare providers. On the other hand, Tinoco-Ojanguren et al. [26] and Khan et al. [33] reveal that expectant women that participated in studies that occurred in Mexico and Bangladesh respectively who had a high-risk pregnancy were blamed for the complications they experienced by other individuals (such as mother’s-in-law and spouses). Roomaney et al. [15], as well as Jackson and Mannix [62] state that the issue of blame may be fixed at mothers from the moment of conception, and continues throughout the pregnancy. Therefore, mothers internalised the ‘mother blaming attitudes’ of the individuals around them and from this blame themselves as opposed to others for the pregnancy-related complications that were often beyond their control. This reflects the argument made by Roomaney et al. [15] and Zahn et al. [57] that self-blame is related to guilt.

#### Mental health disorder

Mental illness during a pregnancy has been receiving much attention lately, but it is by no means a new phenomenon. Various psychiatric disorders have been found to be related to pregnancy. For instance, pregnant women in the included studies expressed symptoms of depression and post-traumatic stress disorder.

##### Depression disorder

This demonstrates that depression is most associated to mothers’ actual experience of motherhood. Yeakey et al. [38], Saukko [23], and Kaye et al. [28] report that participants in different studies conducted in Malawi, England, and Uganda who had a high-risk pregnancy condition (i.e. obstetric fistula, uterine rupture or thrombophilia) suffered from major depressive disorder. As mothers are unable to live up to the idealised expectations of motherhood after experiencing a high-risk pregnancy, they are often left with feelings of guilt and worthlessness that are symptoms of depression. These findings were similar to that of Simmons and Goldberg [14], who reveal that there is an increase in depression disorder for mothers’ after a high-risk pregnancy experience.

##### Post-traumatic stress disorder (PTSD) after the high-risk pregnancy

PTSD was identified as one of the postpartum morbid consequences of a traumatic childbirth. Kaye et al. [28] reveal that participants in a study conducted in Uganda who experienced a traumatic birth were more likely to develop PTSD as opposed to their counterparts. These findings were similar to existing literature. For instance, Ford, Ayers, and Bradley [63] report that 1–6% of women develop postpartum PTSD after birth trauma. Furthermore, another included study conducted by Souza et al. [34] in Brazil indicate that life-threatening events such as maternal near-miss resulted in the participants suffering from PTSD. The above author also state that the women’s experiences of emotional displacement, feelings of blame, isolation, rumination of events, regression, and loss of idealised gestations were all factors that contributed to this disorder. These findings were confirmed by the literature as Polacheck, Dulitzsky, Margolis-Dorfman, and Simchen [64] reveal that expectant women’s emotional cries that occurred during the pregnancy, intense fears of traumatic childbirth and pain were all aspects that are closely associated with PTSD. For this reason, a high-risk pregnancy evokes a range of emotional factors that result in mothers experiencing PTSD.

## Discussion

While pregnancy in general seems to illicit various emotions in women, the findings of this review demonstrated that there are a range of emotional and psychological experiences within a pregnancy at risk. The range of emotions experienced shows that expectant women feel overwhelmed by the adverse diagnosis, being hospitalised and the aftermath of pregnancy. This is because human behaviour is steered by a set of expectations that are socially constructed. Individuals measure themselves against these societal norms of how an experience is supposed to be. For instance, pregnancy is seen to be a straightforward nine-month journey that ultimately leads to the birth of a healthy infant. However, existing literature identifies three ways in which a ‘normal’ pregnancy could be complicated. This includes: 1) abnormal responses of the mother’s body to the pregnancy-induced changes due to pre-existing health problems such as diabetes, high blood pressure; 2) complications that stem from atypical development such as serious abnormalities that occur in the baby; and 3) medical conditions that negatively affect the pregnancy, labour, and delivery. Once a women’s pregnancy is complicated by one of these three conditions, a range of emotional responses may follow the experience of receiving negatively framed information about a pregnancy that could linger into and through the period of hospitalisation for expectant women. Through experiencing these emotions expectant women try to make sense of what is happening to them.

When the pregnant women are hospitalised the experience become disquieting, as the usual adaptation to their pregnancies were disrupted and resulted in experiences of numerous emotions. The aftermath of hospitalisation comes with its own emotional and psychological experiences that continue after the pregnancy. In the long term, women who suffered from a high-risk pregnancy may be diagnosed with depression and PTSD that may result in them having obsessions of child harm, and suicidal thoughts, feeling worthless which places them at risk of either harming themselves or the infant and thus require psychological support [65, 66]. Further to this, children whose mothers suffered with postnatal depression have a high risk of experiencing challenges with handling their emotions and social behavior growing up and it may impact their way of establishing relations with others [67]. Therefore, not all pregnancies are simple, straightforward events and it may negatively affect both the mother and the infant.

By including the emotional and psychological experiences in researching complicated pregnancies it will provide the holistic approach to understanding women and their high-risk pregnancy journey. This will assist medical professionals and mental healthcare professionals to provide competent care and contribute to meeting the needs of the expectant women requiring not only medical support but also psychological support after diagnosis. However, further researchers could include quantitative studies as well as explore the topic further and include more databases that may yield more results and also determining the methodological rigour of several studies. Only two studies made reference to PTSD and depression disorder, which reveals that the majority of the articles regarding women’s emotional and psychological experiences of high-risk pregnancies focus mostly on the emotional aspect and not directing much attention to the psychological part.

### Limitations

With regard to the methodology, this study made use of a meta-synthesis that meant that all findings and conclusions of the study were drawn from the findings of the innovative studies and the understandings that those researchers obtained from their raw data. As a result of this, the researcher had access to the researchers’ inferred data and not raw data, therefore perceived as another limitation of the study. Nonetheless, the researcher of this study took several steps to ensure methodological rigour by including only studies with high methodological quality. Furthermore, the current study has been limited to reviewing articles and studies published between January 2006 and June 2017, which could have affected the amount of articles included in this review as well as the findings of the study.

## Conclusion

This study reviewed 10 databases to explore women’s emotional and psychological experiences of these situations. The review evaluated the literature found on these databases for methodological quality by using three stages of review (i.e. abstract reading, title reading, and full-text reading). These three stages of the review were followed by the utilisation of Noblit and Hare’s [39] process for a successful conduction of the theory explicative of meta-synthesis. This systematic review revealed that women’s experiences are influenced by negative experiences of adverse diagnosis that continue throughout the pregnancy and result in a traumatic childbirth. The childbirth experiences are characterised by poor quality of care due to delay to receive prompt care and negative attitudes of medical staff. The findings of this review are therefore in agreement with previous research in that risks and complications are associated with pregnancy and childbirth and that emotional and psychological experiences overlap the aftermath of high-risk pregnancy in women.

## Data Availability

The study was a systematic review and the information that formed the findings was extracted from the articles listed in Table 1.

## Abbreviations

HELLP syndrome: Haemolysis elevated liver enzymes, low platelet count
HIV: Human immunodeficiency virus
ICU: Intensive Care Unit
NDoH: National Department of Health
PhD: Doctor of Philosophy
PTSD: Post-traumatic Stress Disorder
UWC: University of the Western Cape
